# Cognition in the field: comparison of reversal learning performance in captive and wild passerines

**DOI:** 10.1038/s41598-017-13179-5

**Published:** 2017-10-11

**Authors:** M. Cauchoix, E. Hermer, A. S. Chaine, J. Morand-Ferron

**Affiliations:** 1 Institute for Advanced Studies in Toulouse, Toulouse School of Economics, 21 allée de Brienne, 31015 Toulouse, France; 2 Department of Biology, University of Ottawa, Ottawa, Canada; 3 Station d’Ecologie Théorique et Expérimentale du CNRS UMR5321, Evolutionary Ecology Group, 2 route du CNRS, 09200 Moulis, France

## Abstract

Animal cognitive abilities have traditionally been studied in the lab, but studying cognition in nature could provide several benefits including reduced stress and reduced impact on life-history traits. However, it is not yet clear to what extent cognitive abilities can be properly measured in the wild. Here we present the first comparison of the cognitive performance of individuals from the same population, assessed using an identical test, but in contrasting contexts: in the wild vs. in controlled captive conditions. We show that free-ranging great tits (*Parus major*) perform similarly to deprived, captive birds in a successive spatial reversal-learning task using automated operant devices. In both captive and natural conditions, more than half of birds that contacted the device were able to perform at least one spatial reversal. Moreover, both captive and wild birds showed an improvement of performance over successive reversals, with very similar learning curves observed in both contexts for each reversal. Our results suggest that it is possible to study cognitive abilities of wild animals directly in their natural environment in much the same way that we study captive animals. Such methods open numerous possibilities to study and understand the evolution and ecology of cognition in natural populations.

## Introduction

Wild animals have evolved a given set of cognitive abilities in response to ecological and social constraints present in their natural environment, and ongoing environmental changes are likely to continue the process of natural and sexual selection on a number of cognitive abilities^1,2^. Understanding contemporary selection on cognition requires ecological studies of cognition in which the agents of selection and fitness consequence of individual differences in cognitive performance can be taken into account^3,4^. In order to minimize impact on individual’s life history and fitness, studies on the evolutionary ecology of cognition should ideally be conducted in nature on wild animal populations^3,5,6^, but see^7^.

Cognitive abilities of animals have traditionally been studied in laboratory conditions^8–11^. The principal advantage of using captive animals is to control environmental (e.g. food availability) and internal (e.g. energy reserves) factors that could influence cognitive performance at a given time. In the lab, animals are most often tested in an isolated experimental chamber to minimize social and environmental disturbances and deprived of food and/or water to control for motivation. However, most model species are highly social (e.g. mice, rats, macaques, baboons, guppies, pigeons) and are thus likely to experience stress during isolation with possible consequences on brain function, behaviour and cognition^12–14^. Although animals born and raised in the lab may not experience stress during temporary isolation, wild animals temporarily brought into captivity are particularly likely to experience acute stress under such conditions^15^, which may take several days or weeks before returning to baseline^16^. Acute stress experienced during a cognitive task can bias cognitive performance^17–20^. Additionally, increased motivation through food deprivation can produce cognitive performances exceeding the range normally expressed in nature, or on the contrary, reduce performance, especially in tasks requiring inhibition^21^. These effects are particularly problematic if they produce individual differences in performances that are uncorrelated with those observed in nature^22^.

Over the past decade, an increasing number of studies have tested cognitive performance on semi-free ranging animals in social groups either in zoos or in large parks^8,23^. Interestingly, Gazes *et al*.^24^ have validated this approach by showing that macaque monkeys (*Macaca mulatta*) in large enclosures perform just as well as animals tested in isolated laboratory conditions in a series of cognitive tasks. Moreover, the breadth of species and tasks studied in the wild has increased quickly (see^5^ for a review), and several researchers have begun studying individual difference in cognitive performance in free-ranging individuals in the wild^25–29^. However, the perceived lack of control on potential confounding variables continues to slow the development of studies on cognition in the wild. Individual motivation and social context vary in natural conditions and thus can add noise to cognitive performances measured. Moreover, most cognitive paradigms rely on appetitive tasks and the multitude of alternative feeding sources available to animals in the wild could be expected to reduce participation rates in a given cognitive task.

This trade-off between tight control of individual condition and a more natural setting has presented us with a gap between lab and field cognition studies that slows down progress in understanding how cognition evolves. In this paper we aim to build a bridge between new studies on cognition in the wild that enable us to understand the ecology and evolution of cognition but where tests are poorly controlled, and experiments run in captivity that are well-controlled but where performances might be decoupled from those expressed in natural settings. In particular, we are faced with two challenges: (i) we do not know if cognition can be measured as accurately in the wild as it is in the lab and (ii) we have little evidence of whether cognitive abilities recorded in captivity are generalizable to a wild and natural context.

We ran the same cognitive task on free-ranging great tits (*Parus major*) foraging in variable group sizes in the wild and on great tits from the same population brought into temporary captivity and tested in small groups after traditional habituation and deprivation procedures. The task was carried out on automated portable operant devices that enable recording of a large number of trials from many individuals simultaneously^27^. We used a serial spatial reversal learning task, in which individuals have to inhibit a previously rewarded response to learn a novel stimulus-reward association^30,31^. In comparative psychology, a large number of species have been tested using similar reversal learning tasks since the early 1960s^32–34^. Particular attention has been paid to the improvement in performance over successive reversals^35,36^ which has been considered as a hallmark of ‘intelligence’^32^ and suggested as a good marker of general cognitive abilites evolution across species^37,38^. Additionally, serial reversal learning tasks rely on quick adjustment in response to repeated changes in stimulus-reward associations, and thus could be a useful tool to understand how animals adjust their behaviour to rapidly changing environments^30,39^.

Our aims were to validate an experimental procedure to test reversal learning ability of wild birds, compare performance of free-ranging birds voluntarily participating in trials in the field versus in more traditional conditions (controlled test duration and food deprivation), and investigate improvement of performance over successive reversals in wild and captive individuals. Performance of birds in captivity and in the wild should mainly depend on the relative impact that the stress of captivity and the lack of control on internal (e.g.: absence of food deprivation) or external (e.g.: large group) factors in the wild has on cognitive performance. If captivity induces very high stress levels, then we would predict weaker participation and lower performance for captive birds than wild birds. On the other hand, alternative food sources in the wild or high competition could reduce motivation to participate in tests or disturb learning processes and we would predict lower participation and performance for wild birds relative to captive birds. Equivalent performance in the two settings could result if captive birds experience high stress levels and wild birds are strongly affected by the availability of other food sources or competition at operant boxes, leading to low performance in both cases; or if captive birds experience low stress levels and wild birds have few reliable alternative food sources leading to high participation and performance in both cases.

## Methods

### Population and general procedure

We studied wild great tits (*Parus major*) at 6 sites near Moulis, France. At capture, birds were equipped with a passive integrated transponder (PIT-tag; IB Technology, UK) ring enabling them to interact with fully automated operant boxes^27^. After recording of morphometric measures, birds were either directly released at their capture location for field experiments, or brought to aviaries at Moulis within less than 2 hours after capture for use in captive experiments. Each site was either used as a source for captive animals or for experiments in the wild, but not both, in order to preserve natural social structure in wild groups.

Experiments in the wild were conducted at two separate sites (~3.5 km, M1: 42.96°N, 1.08°W, C1: 42.94°N, 1.04°W) over 39 days from end of January to beginning March 2015. To reduce the effects of strong competition or monopolization of operant boxes by dominant individuals we set-up two operant boxes per study site. Boxes were synchronized by radio transmission of data and thus a bird could start the task on one box and continue on the other one at the same point of the learning program^27^. 

For experiments in captivity, we captured and tested birds from 4 different sites (Au: 42.96°N, 1.10°W; Le: 42.98°N, 1.11°W; Cs: 42.93°N, 1.12°W; An: 42.93°N, 0.91°W, 2.5–16 km from wild sites) between February and March 2016. While sites for testing wild birds are at the same elevation, captive birds came from two different elevations for the purposes of another experiment and we thus control for the effect of altitude in all statistical models. Captive birds were housed in a group composed of 2 to 5 individuals from the same capture location in 1 × 4 × 3 m (w × l × h) aviaries and allowed to habituate to captive conditions for one week (food at libitum, minimal human disturbance and shaping with dummy operant boxes). Birds had the opportunity to conduct the reversal learning task on one operant box twice a day during a period of 2 hours at each session (8 h–10 h and 14 h–16 h) following food deprivation. Morning tests were conducted one hour after sunrise with food removed the night before whereas afternoon tests directly followed a single hour of food deprivation. Birds were fed at libitum with seeds and mealworms between learning sessions and released at their capture sites after 3 weeks of captivity.

### Reversal learning task

A week before the experiment started, both in the wild and in captivity, birds were shaped to use operant boxes. We used dummy wooden operant boxes providing food at the location of future pecking keys (a mixture of butter and seeds) and reward hole locations (sunflower seeds). Reversal learning task was then run using operant boxes described by^27^. These apparatuses are equipped with an RFID antenna to automatically recognize marked individuals, have 2 transparent keys aligned horizontally to both display stimuli (LED light) and collect responses (bird pecks), and a rotating wheel behind a feeding hole to deliver a single reward. The learning program was individualized based on RFID tags such that social learning or copying of responses was unlikely better than a random response. In the wild, birds were rewarded with a single sunflower seed for each response, while captive birds were not willing to complete the task for seeds during pre-trials and were thus rewarded with a half of a dry mealworm.

**Figure 1:**
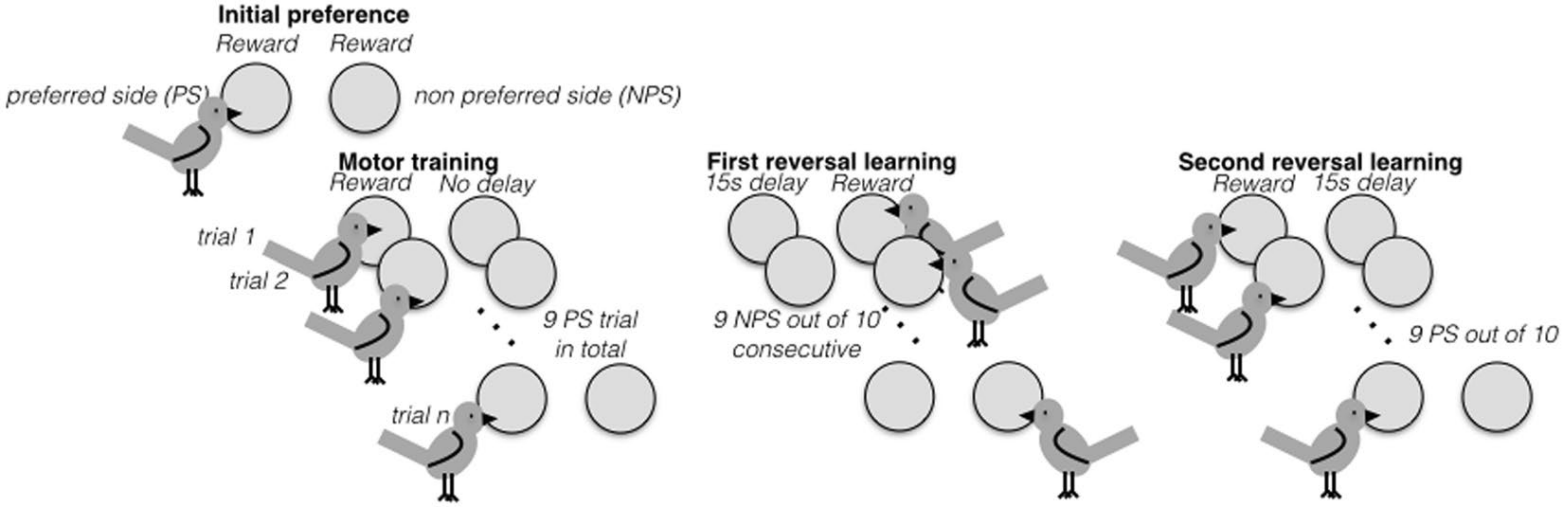
Graphical representation of the experimental protocol. In this example, the bird chooses the left side in the preference task and is then rewarded on the left side (preferred side) during the motor training phase. After a total of 9 correct trials of motor training, it starts the first reversal learning in which it is rewarded on the right side (non-preferred side) and punished on the left side. After reaching the learning criterion (9 correct response out of 10 consecutives trials), the bird then shifts to the second reversal in which it is rewarded on the left side and punished on the right side. Successive reversals are allowed to continue until 99 reversals in captivity and until 48 reversals in the wild. Artwork by M. Cauchoix.

Birds were engaged in a successive spatial reversal-learning task in which they had to choose between a left and a right key by pecking it (Fig. 1). During the entire experiment (except when the rewarding wheel was empty or a bird was punished) the two side keys were lit with a white LED.

For each bird the task started with an initial spatial preference test. The first key pecked (right or left) was assigned as the preferred side for that bird and this peck was rewarded. Immediately after this initial choice we reinforced this preference and provided further opportunities for motor training by rewarding 9 times a response to the preferred side. During this motor training phase, response to the non-preferred side did not deliver food but was not punished (lights were only turned off for 500 ms) to not discourage birds in this early stage of the experiment. Once an individual accomplished 9 successful trials in total, the spatial reversal learning task began on the non-preferred side such that the initially preferred side was no longer rewarded but the non-preferred side was rewarded. Birds had to reach the learning criterion of 9 correct responses over 10 consecutive trials to advance to the next reversal learning stage in which reward contingencies were again switched spatially. A trial is defined as an individual pecking one of the two keys.

During reversal learning a correct response resulted in a single reward delivery while an incorrect choice resulted in the lights going off behind both keys, as well as a period during which the box was unresponsive to that specific individual either for 15s or until another individual visited the box, whichever occurred first. In the wild, individuals were allowed to participate in up to 48 reversals and then were blocked from further use of the operant box to allow other birds to participate in the task. In captivity, reversals continued until the end of the two week experimental period or 99 reversals were reached.

**Figure 2:**
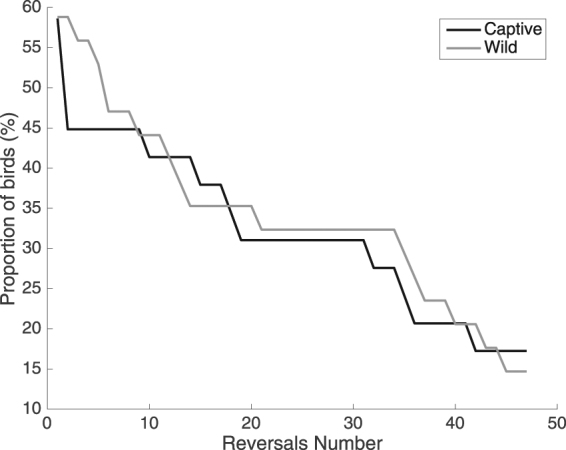
Proportion of individuals successful in each reversal computed over the total number of birds who were logged on a device at least once. Captive birds are in black and wild birds are in grey.

We computed success rate in the different stages of the task as the number of birds succeeding in a given reversal divided by the total number of birds that were logged at least once by the apparatus (Fig. 2). We used this denominator since in the wild capture began 4 months before identification at operant feeders and a number of birds could thus have disappeared from the study site due to death or movement. Using the total number of individuals banded for this ratio would not represent the number of birds potentially present at the study site when the experiment started and thus would not be comparable with the captive experiment.

We measured reversal learning performance in this task using several variables. At the trial level (right or left key peck), we measured *single response accuracy*: 1 if the response is correct and 0 if incorrect. Within a reversal stage we computed *sliding window accuracy* as the number of correct responses divided by the total number of responses in 10 consecutive trials across a sliding window (i.e.: sliding window accuracy is computed for each trial on the 10 previous trials) to obtain learning curves. For each reversal stage we also computed the *reversal accuracy* defined as the total number of correct responses divided by the total number of responses in a given reversal rather than across a sliding window of 10 consecutive trials. We also calculated the *trials to criterion* (TTC) as the total number of trials (sum of correct and incorrect responses) needed to reach the learning criterion.

All our methods were carried out in accordance with published guidelines of the Animal Behavior Society, Association for the Study of Animal Behaviour, and the Ornithological Council. All experimental procedures were conducted under permits to A. Chaine from the French bird ringing office (CRBPO; n°13619) and animal care permits from the state of Ariège (Préfecture de l’Ariège, Protection des Populations, n°A09-4 for experimental procedures and n°A09-321 for holding birds in captivity) and the Région Midi-Pyrenées (DIREN, n°2012-07).

### Statistics

We used Pearson’s Chi-squared test with Yates’ continuity correction and contingency tables to test for differences in the proportion of birds using the device, performing each experimental step of the task in both environments or sex and age bias in each of these proportions. We used a log-rank test to compare “survival curves” corresponding to success of birds in each stage of the task (Fig. 2).

For each experimental context (wild and captive), we first evaluated learning of the task within each reversal and across reversals. We tested if trial and reversal number predicted *single response accuracy* using a binomial GLMM with individual as a random intercept, and fixed effects of study site for wild birds or cage group size and elevation for birds in captivity. We further evaluated performance improvements over successive reversals by testing the effect of reversal number on *reversal accuracy* and on *TTC* of each reversal using LMM with the above-mentioned random and fixed effects. To examine the difference in reversal learning performance in the wild and in captivity, we first compared performances (trial accuracy and TTC) for each reversal using independent t-tests. We corrected for multiple comparisons using Bonferroni correction upon detection of p-values < 0.05. Finally, to investigate if performance improvements over reversals differed in captivity and in the wild, we tested the interaction between experimental context and reversal number on reversal accuracy and TTC using LMM with individual as a random intercept. All data processing was performed using a commercial software package (MATLAB 8.6, The MathWorks Inc., Natick, MA, R2015b) and GLMMs were fit using the statistical package *lme4* ^40^ in *R* (version 3.4.0, R Core Team 2017).

### Data availability

upon request.

## Results

### Participation in the task

In the wild, 63% (34 out of 54) of locally-banded great tits perched at least once on the device’s antenna whereas 100% (29 out of 29) of captive birds perched on the antenna (Table 1). Sex (Contingency table: χ^2^ = 0, df = 1, P = 1) and age (Contingency table: χ^2^ = 0.52, df = 1, P = 0.41) ratio of birds logged was not different from the locally-marked population for the wild experiment and was not calculated for captive birds as all birds perched on the device (SI Tables 1, 2 and 3).

**Table 1:** Number of birds completing each step of the experiment: individually identified, logged on the apparatus, passing motor learning task and completing at least 1, 10, 30, 48 or 79 reversals.

	Banded	Logged	Motor	1 R	10 R	30 R	48 R	79 R
Wild	54	34	24	20	15	11	5	/
Captive	29	29	26	17	12	9	5	1

Note that in the wild the program only allowed birds to perform up to 48 reversals.

We further investigated how many of these birds passed initial shaping steps of the task among birds that visited the device at least once (logged). The proportion of birds pecking a key at least once (Initial preference task) was very similar (97% (33/34) in the wild, 100% (29/29) in captivity; Contingency table: χ^2^ = 0.03, df = 1, P = 0.86) as was the proportion of birds succeeding in the motor training task (70% (24/34) in the wild, 90% (26/29) in captivity; Contingency table: χ^2^ = 0.40, df = 1, P = 0.53).

We then tested if participation in the reversal-learning task (proportion of birds completing at least the 1^st^ reversal for all birds logged on the apparatus) was different in captivity or in the wild (Table 1) and biased by sex or age (SI Table 4). Participation in the cognitive task was highly similar in both contexts with 59% of all birds that contacted the apparatus completing the first reversal in both conditions (20/34 in the wild, 17/29 in captivity, Table 1, Contingency table: χ^2^ = 1.31, df = 1, P = 0.25). In both contexts, participation in the reversal was not affected by sex (Contingency table: Wild,: χ^2^ = 0.02, df = 1, P = 0.88; Captivity: χ^2^ = 0,66, df = 1, P = 0.42) nor by age (Contingency table: Wild,: χ^2^ = 0.24, df = 1, P = 0.61; Captivity: χ^2^ = 0.25, df = 1, P = 0.62).

We further evaluated if for each reversal (up to the maximum in the wild: 48^th^) the proportion of birds succeeding in the reversal differed between the wild and captivity (Fig. 2). No significant difference was found for any reversal (Contingency table: minimum P = 0.49, χ^2^ = 0.47, df = 1 in reversal 2). Finally, a log-rank test that compares two survival functions was used to compare the number of birds participating in each reversal (i.e. “surviving” to the next reversal) between the wild and captivity and showed no significant difference between the two contexts (UL = 3.32, z = 0.80, P = 0.42).

### Reversal learning performance

#### First reversal

**Figure 3:**
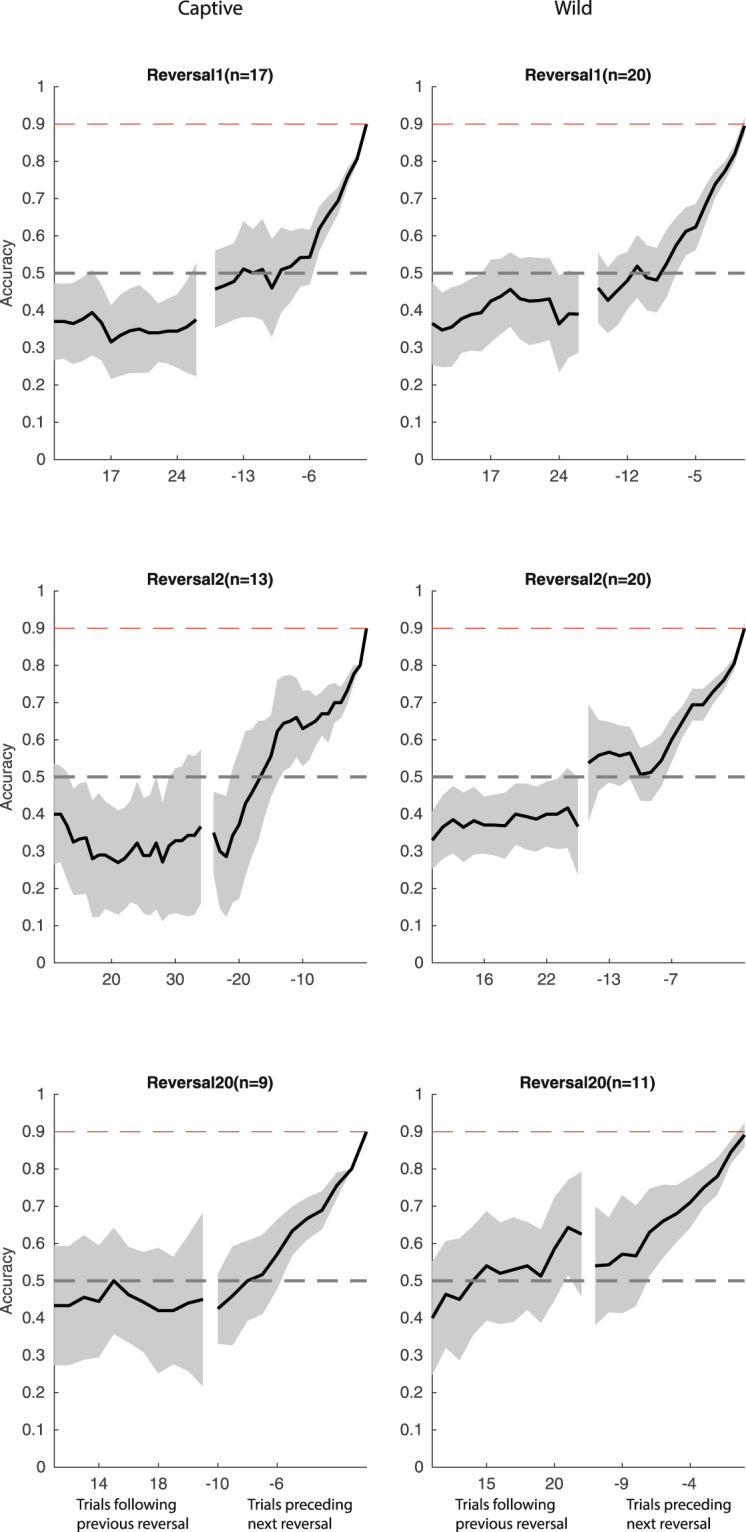
Learning curves in first (top panel), second (middle panel) and 20^th^ reversal (bottom panel) for captive (left panel) and wild birds (right panel). For each graph, the black line and grey shaded area represents *sliding window accuracy* (+/−95% bootstrapped CI) computed on a 10 trial sliding window and is plotted for early trials directly following previous reversal (left part of the curve) and late trials preceding next reversal (right part of the curve). Learning criterion is reached when a bird performed 9 correct trials out of 10 successive trials (0.9; top dashed line). As birds required a different number of trials to reach the learning criterion, learning curves are plotted only for trials performed by at least half of the birds who completed that reversal, aligned on beginning (trials following previous reversal, positive value on x axis) or end (trials preceding next reversal, negative value on x axis) of the reversal session. Middle dashed line represents chance (0.5).

Average learning curves representing *sliding window accuracy* computed on a 10-trial sliding window show the dynamic of improvement in accuracy in each context (Fig. 3, top panel). In both environments, birds successfully learned to inhibit pecks at the previously rewarded key and used the other key. There was a significant, positive effect of trial number on single response accuracy in both captivity (binomial GLMM: df = 16645, z = 30.1, P < 0.0001) and in the wild (binomial GLMM: df = 19274, z = 24.4, P < 0.0001).

#### Improvement of performance over all reversals

**Figure 4:**
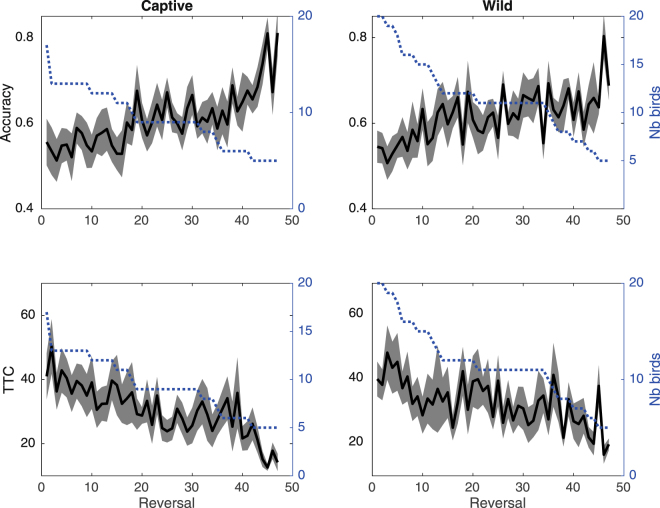
Reversal accuracy and TTC (black line +/−95% bootstrapped confidence interval in grey) according to successive reversals for captive (left panel) and wild (right panel) birds. The dotted line indicates the number of birds involved in each reversal (right y-axis).

Both wild and captive birds improved their reversal accuracy (LMM, captive: df = 523, z = 9.5, P < 0.0001; wild: df = 545, z = 6.5, P < 0.0001) and diminished the number of trials needed to reach the learning criterion (LMM, captive: df = 523, z = −6.5, P < 0.0001; wild: df = 545, z = −3.5, p = 0.0005) along successive reversals (Fig. 4). At the trial level, the proportion of correct responses also increased according to reversal number in captivity (binomial GLMM: df = 16645, z = 13.4, P < 0.0001) and in the wild (binomial GLMM: df = 19274, z = 8.7, P < 0.0001).

#### Influence of experimental conditions on reversal learning performance

We first tested if reversal accuracy and TTC differed between experiments conducted in the wild or in captivity. No significant difference was found: for all reversals except the 46^th^, p-values using a t-test are >0.05. An apparently significant difference was found both for reversal accuracy (t = 3.4; df = 8; P = 0.009) and TTC (t = −2.9; df = 8; P = 0.02) in the 46^th^ reversal but this difference did not hold with correction for multiple comparisons.

Finally, performance improvements over reversals were not significantly different either when considering accuracy (LMM, interaction of reversal number and experimental context: t = −0.35, df = 1068, P = 0.97) or the trials until the learning criterion was reached (LMM, interaction of reversal number and experimental context: t = 0.46, df = 1068, P = 0.65).

## Discussion

Measuring cognition in the wild presents many advantages relative to captivity^5^ and is critical for a complete understanding of how cognition evolves^3,4,6^. For example, measurements in the wild allow quantification of socio-ecological determinants of cognitive variability, may reduce stress, and minimize the impact of experimental procedures on posterior life-history traits and fitness. However it is still unclear whether it is possible to measure cognitive performance in the wild in a way that returns similar assessments as in more traditional, controlled conditions in captivity. Here we show that free-ranging great tits voluntarily participating in reversal learning trials in the field performed similarly to captive and deprived birds from the nearby populations, as measured by similar participation rates, reversal learning performance, and improvements in accuracy and trials to criterion over successive reversals.

Obtaining sufficient sample sizes of cognitive performance in the wild is challenging and can limit cognitive research in free-ranging populations. In wild populations, the proportion of individuals interacting with an experimental apparatus and successfully completing a cognitive task is generally lower than the 59% reported here, ranging from 0.5% to less than 50% (e.g.: great tits, hyena, vervet monkeys, meerkats)^41^. However, participation rate strongly depends on what population and species is considered. In some cases, participation rate may be low because the marked population includes transients, individuals from a different part of the study site, or attrition due to death. Quantifying participation rates on the number of animals contacting the apparatus, as opposed to all potential subjects in the population^27,42^, allows estimating success of an experiment at recruiting participants amongst those that are still present. This value is more comparable to values presented in laboratory studies, as individuals who do not interact with the apparatus or die before the experiment begins are generally not reported. While this measure may miss some individuals who are present but do not interact with the device, it has the advantage of reducing differences between studies and making results more comparable. Populations or species may differ in the proportion of the population that interacts with the device, in their degree of mobility, and the delay between marking animals and the start of the experiment likely increases attrition. Basing task participation rates on the population that is present at the start of the experiment either by direct observation or logging on the device more accurately reflects the resident population and allows for a standardized comparison across studies.

Participation also likely depends on a species’ social system and timing of the study. For instance, cognitive testing of wild territorial birds during the breeding season has led to very high participation rates ranging from 67% to 100%^26,29,43,44^. However, in such systems, participation might be biased toward one sex^26,29^. Furthermore, installation of testing devices on individual territories is time consuming over a short critical period (part of a breeding season) and therefore can seriously constrain sample sizes or the complexity of tests. It is noteworthy that our study, despite using a mobile species forming roaming fission-fusion flocks during winter^45^, has returned a very similar proportion of individuals successfully completing each successive reversal in the field and in captivity. However, increases in task complexity are likely to limit sample sizes, potentially limiting the use of more complex tasks in the wild. Indeed, the proportion of birds completing 10 reversals dropped from 59% to 44% of logged birds in the wild and 42% in captivity. Such numbers can provide guidelines for estimating sample size in future studies and be used to limit the complexity of repetitive tasks such as serial reversals.

Individual determinants such age, sex, neophobia or body condition can also strongly affect the propensity to participate and complete a cognitive task (see ref.^41^ for a review). We did not find an age or sex bias in the proportion of birds interacting with the device or completing the first reversal either in the wild or in captivity. However, other individual characteristics such exploration rate^46–50^, dominance^46^, neophobia^51^ or body condition^41,52^ that were not measured here, could potentially affect performance of wild and captive bird. Regardless, the lack of age and sex bias in our results suggest that measurement of wild birds in mixed flocks outside of the breeding season has the ability to capture a demographically representative sample of the population.

Manually set-up tasks only allow a single test of a single individual before intervention by the observer. Such an approach limits the number of individuals that might participate, the number of trials per individual for a given observer effort, and the complexity of multi-session learning-related tasks in contrast to studies in captivity. In some cases, the total number of individuals tested will not be sufficient to reveal conclusive relationships between cognitive abilities and socio-ecological or fitness variables^26,53,54^. Here, using automated operant devices^27^, we recorded over 25000 trials in the field on 33 individuals in less than 40 days. Furthermore, automation of the task allowed some birds to perform up to 48 successive reversals in a learning task providing data to show improvement of performance over successive reversals for the first time in the wild. Automation is critical to measure trial-intensive cognitive performance and is indeed largely used in comparative cognition in captivity^23,24,55–59^. There is no doubt that cognitive ecologists interested in measuring complex cognitive functions in the wild or wild psychometrics^6^ will benefit from automated cognitive testing so as to reach comparative cognition standards^3^.

A concern for testing cognition in the wild is that there is little control over external factors that could influence motivation or learning performance relative to traditional tests in captivity. Our reversal learning testing approach is similar to recent work in comparative cognition on semi-free ranging primates^23,24,60^ based on “voluntary” cognitive testing. In these studies, individuals have the option to participate in cognitive testing at any moment in time, and are not food-deprived before test periods. This procedure assumes that voluntary participation is achieved past a given threshold of motivation, although the quantitative relationship between participation, motivation level, and cognitive performance at the individual level remains to be investigated. High participation rate, as well as similar reversal learning performance and improvement over successive reversals between our free-ranging and our deprived, captive great tits suggest that motivation levels may be similar in both contexts. It also suggests that learning rates are reasonably robust to the ecological context and to various sources of environmental noise. For example, similarly high levels of participation and performance between captive and wild birds suggest that the stress of temporary captivity did not play a large role in cognitive performance. Likewise, a lack of food deprivation did not limit wild birds from participating in tasks and may only be important in captivity where ad libitum high quality food is available. Taken together, our results suggest that recording reversal-learning performance of wild or captive birds seems equally suitable. Measurement of the same individuals in both wild and captive contexts in future studies could reveal individual variation in how stress and food deprivation impact cognitive performance and complement the population-wide patterns shown here.

Successive spatial reversal learning tasks^30,31^ are analogous to ecological scenarios where the association between a food resource and an habitat feature changes over time and therefore provides a relevant tool to compare cognition between captive and wild birds. During reversal learning, subjects first have to learn a reward-stimuli association (e.g. a resource on one tree species), then subsequently must inhibit this previously learned association and learn a new association (e.g. resource on a different tree species). Key cognitive abilities supposedly required to succeed in this task range from spatial associative learning, to executive functions such as inhibitory control (i.e. the ability to inhibit a prepotent response)^61^ or cognitive flexibility (i.e. ability of subjects to attend to a shift in reward contingencies based on stimulus dimension, and to adapt their behavior in response to that shift)^62^. Here we show that the performance of great tits improved across successive reversals, thereby providing the first demonstration of such improvement over serial reversals in the wild. The cognitive mechanisms allowing such improvement in performance are more elusive and go from simple mechanisms such as decreasing the inhibitory effects of previously learned patterns, to rule learning (e.g. after 9 successful choices, the pattern is likely to switch)^30,61,63,64^. Interestingly, Bonté *et al*.^62^ tested the same baboons both in successive reversal learning and in tasks aimed at measuring inhibitory control and cognitive flexibility independently. They found that an increase in performances across successive reversal learning was positively correlated with cognitive flexibility tasks but such an association was not present for inhibitory control tasks. More work from comparative psychologists is needed to clearly identify to what extent and during which step each cognitive function plays a role in successive reversal learning (but see ref.^61^). Comparative studies have shown that species facing high variability in food supply^65^, complex social environments^30^ or variation in foraging strategies^66^ show higher performance in reversal learning than similar species living in more stable and predictable environments. Measuring inter-individual differences in reversal learning in the wild will enable us to better understand how natural selection operates on related cognitive abilities in species faced with rapidly changing environments^1,2^.

## Conclusion

Despite increasing interest in cognition of wild animals, studies on free ranging animals remain rare^3,5^. The scarcity of such studies is most likely due to concerns for a lack of environmental control and limited sample sizes in the wild. Here we used a recently developed field-portable device^27^ to show that performance in a classical spatial reversal learning task of wild free-ranging great tits was similar to that of individuals brought to captivity and motivated through food deprivation. While our analysis focused on mean population patterns of captive vs wild animals, the same methods could be used to examine variation among individuals in reversal learning in wild populations. In doing so we hope to convince people familiar with laboratory experiments and interested in the ecology and evolution of cognition that a complementary avenue of research is possible through direct experimentation in the natural habitat, even though not every model species or cognitive tasks might be equally suitable. We also hope that developing cognitive tasks for wild animals, such as successive reversal learning, will help improve our understanding of the evolutionary ecology of cognition.

## Electronic supplementary material

Supplementary Information 
