# Supplementary material for: Cognition in the field: comparison of reversal learning performance in captive and wild passerines
Source: Sci Rep. 2017 Oct 11;7:12945. doi: 10.1038/s41598-017-13179-5 (PMC5636806; doi:10.1038/s41598-017-13179-5)
Supplement: Supplementary file 1 — Supplementary Information [file 41598_2017_13179_MOESM1_ESM.pdf]

Supplemental information

Cognition in the field: comparison of reversal learning performance in captive and wild passerines.

Cauchoix M.[1], Hermer E.[2], Chaine A.S.[1,3], Morand-Ferron J.[2]

Table1: Descriptive statistics of the birds marked for the experiment in the wild. Shown is the number of males (females not shown), and juveniles (adults not shown)

Site	Total	Male	Juvenile
**Moulis**	27	14	19
**Cescau**	27	13	19

Table2: Descriptive statistics of the birds captured for the experiment in the captivity

Site	Total	Male	Juvenile
**Andy**	5	3	5
**Cap de sour**	11	4	11
**Aubert**	9	9	4
**Ledar**	4	3	3

Table3: Descriptive statistics of logged birds (perched at least once on an apparatus)

Site	Total	Male	Juvenile
**Wild**	34	17	19
**Captive**	29	19	23

Table4: Descriptive statistics of birds participating in the reversal learning task (completed at least the 1st reversal)

Site	Total	Male	Juvenile
**Wild**	20	12	14
**Captive**	17	9	15
